# Coping with stressful life disruptions due to long COVID: A qualitative study

**DOI:** 10.1371/journal.pone.0329831

**Published:** 2025-08-19

**Authors:** Marina B. Wasilewski, Jaylyn Leighton, Logan Reis, Abirami Vijayakumar, Mahala English, Kris Sanchez, Sander L. Hitzig, Michelle L.A. Nelson, Christine Sheppard, Lawrence Robinson, Rosalie Steinberg, Melody Nguyen, Mark Bayley, Nick Daneman, Charissa Levy, Susie Goulding, Chester Ho, Robert Simpson

**Affiliations:** 1 Sinai Health Science of Care Institute; Institute of Health Policy, Management and Evaluation, Dalla Lana School of Public Health, Toronto, Ontario, Canada; 2 St. John’s Rehab Research Program, Sunnybrook Research Institute, Sunnybrook Health Sciences Centre, Toronto, Ontario, Canada; 3 Factor-Inwentash Faculty of Social Work, University of Toronto, Toronto, Ontario, Canada; 4 Sunnybrook Health Sciences Centre, Toronto, Ontario, Canada; 5 UHN Toronto Rehabilitation Institute, Toronto, Ontario, Canada; 6 Sunnybrook Research Institute, Toronto, Ontario, Canada; 7 University Health Network, Toronto, Ontario, Canada; 8 Division of Physical Medicine and Rehabilitation, Department of Medicine, University of Toronto, Toronto, Ontario, Canada; 9 Division of Physical Medicine & Rehabilitation, Department of Medicine, University of Toronto Toronto, Ontario, Canada; 10 Faculty of Medicine & Dentistry, University of Alberta, Edmonton, Alberta, Canada; National Institute of Demographic Studies: INED, FRANCE

## Abstract

**Background:**

Long COVID impacts people’s physical health and cognition which immensely affects their psychosocial well-being. A larger study was conducted that explored the psychosocial impacts of Long COVID on individuals and caregivers. This paper focuses on the impact of these stressful disruptions on one’s health and psychosocial well-being, and how individuals cope with them.

**Methods:**

Utilizing a qualitative descriptive approach, we conducted interviews with 67 participants (52 people with Long COVID (mean age: 50.4) and 15 caregivers (mean age: 50.0)). People with Long COVID and caregivers were recruited from healthcare institutions through care team referrals, patient partners, and health organizations and via social media platforms. A thematic codebook developed through inter-coder agreement processes was used to analyze the data.

**Findings:**

Three key themes were identified: (1) Disruptions in people with Long COVID and caregivers’ lives are characterized by a deviation from their perceived ‘normalcy’, (2) Disruptions lead to substantial stress, loss and grief (independence, agency, meaning, and purpose), and (3) People with Long COVID and caregivers cope with stressful disruptions by adapting their daily activities.

**Conclusion:**

Our findings make the case for supportive rehabilitation strategies that address the psychosocial repercussions of Long COVID to help mitigate feelings of loss and grief, thereby increasing individuals’ overall quality of life and well-being.

## Introduction

Emerging evidence indicates that 10–35% of people infected by COVID-19 will experience prolonged and debilitating symptoms long after the acute stage of infection. [1–3] In Canada, this could be up to 1.64 million individuals who have experienced ‘Long COVID’ symptoms to date. Long COVID is understood to be “a collection of symptoms that develop during or after a COVID infection (either confirmed or suspected) and that continue for more than 12 weeks”. [2,4] Long COVID entails fluctuating symptoms that can impact several organs and bodily systems (e.g., nervous system (brain and nerves), respiratory (lungs), circulatory (heart), and digestive (stomach and colon) at once and for a prolonged period of time. [5,6] People with Long COVID (PWLC) commonly report symptoms such as severe fatigue, headache, brain fog, and muscle aches and pain, which negatively affected their mental health. [2,3,7–13]

PWLC face widespread challenges that disrupt their daily lives as Long COVID creates uncertainties about their futures, leading to abrupt changes in their perceived roles and identities. [14,15] Many PWLC report that their symptoms – most notably cognitive impairments such as brain fog and the exhaustion from fatigue– prevent them from carrying out daily activities and returning to work, domains of life that are core to one’s sense of identity and can provide a sense of mastery and self-efficacy. [16,17] For example, PWLC describe questioning their sense of self when they are unable to carry out their regular routines, especially when these roles and activities are considered to be integral aspects of their identity. [18] Many people who experience a sudden onset of illness or impairment also experience similar forms of psychosocial stress associated with the incurred disease or trauma, as such, those experiencing Long COVID symptoms are no exception. [19] Furthermore, the lack of recognition of the serious impact of Long COVID as a health condition for a plethora of reasons (e.g., novel condition, one’s medical status, lack of diagnostics, unknown phenomenology, and abundance of documented symptoms), has left many people feeling misunderstood and invalidated by healthcare professionals and organizations, family, friends, co-workers, and employers. [4] In addition to these challenges, PWLC must contend with an uncertain prognosis and the question of whether their lives will ever return to normal. [18] Living with Long COVID can have serious consequences for mental health and well-being as people struggle to make sense of their experiences and adjust to their reduced capacity for functioning in most, if not all, aspects of their daily living. [14,15]

This loss of identity has similarly been reported in people suffering from other chronic conditions with similar symptoms to Long COVID. [16,18,20] For instance, Myalgic Encephalomyelitis/Chronic Fatigue Syndrome (ME/CFS) patients with similar reports of fatigue, sleep disturbances and cognitive impairment also report difficulties carrying out their daily routines and worsening mental health (e.g., anxiety). [21] For the ME/CFS population, identity disruption has been associated with reduced confidence and self-esteem and an increased dependency on others, such as caretakers. [21,22] Additionally, those with ME/CFS often see a decrease in ability to maintain a highly active lifestyle, which can generate a sense of loss of their past selves and a decreased sense of personal agency. [23] These observations strongly suggest that PWLC will also face disruptions in their lives due to the chronic, episodic and eminently disabling nature of the condition. Yet, the scope and impact of these disruptions in the context of Long COVID has not yet received widespread attention.

The experience of caregiving for a person living with a chronic condition is different than that of the “patient” as they feel the basic structure of disability through disruptions in their own lives rather than through physical or cognitive symptoms. [24] Given the interconnected nature of family relations and the vital role caregivers play in the home, the experience of family/friends (caregivers) is a key knowledge gap in Long COVID rehab. Long COVID care and support initiatives have been limited to date in their consideration of how disruptions are co-experienced by PWLC and caregivers (e.g., shifts in their relational dynamic) and which disruptions may be common or unique to each group.

The present paper aimed to explore the disruptions experienced by PWLC and caregivers, their impact, and how PWLC and caregivers respond to and cope with them. Specifically, we aimed to:

1. Explore the nature and extent of disruptions that PWLC and caregivers experience and the impact they have on health and well-being.
2. Understand how PWLC and caregivers cope with and adapt to the stress of these disruptions.

## Materials and methods

This paper draws on data from a broader study aimed at co-designing rehabilitation supports for the psychosocial aspects of living with Long COVID. [25] The first phase of this project included qualitative interviews with PWLC and caregivers, to explore the broad range of experiences associated with living with Long COVID and navigating the Canadian healthcare system. A qualitative descriptive approach was employed to represent data in the participants’ own words as they made sense of their lived experiences, thereby making results more meaningful and relevant for justifying actionable change. [26,27] In this paper, we only report on data from PWLC and caregivers that specifically addresses the proposed research questions, data from Health Care Providers were excluded.

### Participants and recruitment

To be included in the study, individuals had to self-identify as: a) having either a confirmed case of COVID (i.e., positive COVID nasal swab or antibody test) or suspected case of COVID (i.e., a constellation of symptoms and signs consistent with acute COVID followed by prolonged recovery); b) were experiencing prolonged symptoms for a minimum of 12 weeks; c) were able to read, write, and communicate in English; and d) were community-residing adults living in Canada. Thus, those in the acute phase of illness were excluded. Caregivers were included if they were supporting an individual that met the above criteria and they themselves could read, write, and communicate in English. All caregivers included in the study were family caregivers to a PWLC (spouse, partner, or parent).

PWLC and their caregivers were recruited primarily online through a large national peer support group for long COVID (“COVID Long Hauler Support Group Canada” Facebook page). We also recruited PWLC and caregivers using social media platforms (e.g., Twitter and Facebook) via the research team’s accounts, our patient partner, as well as various regional, provincial, and national health and social care organizations.

### Data collection

This study was approved by the Research Ethics Board (REB) of Sunnybrook Research Institute and written informed consent was obtained prior to data collection. We created a semi-structured interview guide to explore individuals’ experiences with navigating Long COVID and offering opportunities for them to reflect on ways Long COVID has disrupted their lives (see Table 1 for a list of sample questions). One-on-one interviews with participants were conducted by trained qualitative researchers (ZS, JH, JL) via Zoom or phone. The length of the interviews ranged from 21 to 82 minutes and were carried out between April 4, 2022 and January 19, 2023. Data was collected until saturation of ideas was reached (i.e., no new information collected from subsequent interviews). The audio recordings were transcribed verbatim and any identifying information was removed before being imported into a qualitative coding software (NVivo 12) for analysis. [28] The data that support the findings of this study are available from the corresponding author, Dr. Marina B. Wasilewski upon reasonable request.

**Table 1:** Sample interview questions.

Sample People with Long COVID Interview Questions:	Sample Caregiver Interview Questions:
Could you start by telling me a little bit about your COVID-19 journey? After the onset of COVID symptoms (of after being diagnosed), what were your top concerns and needs? What has helped you navigate day-to-day life while experiencing Long COVID? What has challenged your ability to navigate day-to-day life with Long COVID? What types of things do you feel you need help or support with?	To start, could you tell me a little bit about you and your loved one’s COVID-19 care journey? After your loved one was diagnosed with or experiencing presumed COVID-19, what were your top concerns and needs? What has life been like for you as you’ve supported your loved one with Long COVID? What has challenged you and your loved one’s ability to navigate day-to-day life with Long COVID? What types of things do you feel you need help or support with?

### Data analysis and analytic rigor

We used a codebook thematic analysis approach guided by several works of Braun and Clarke [29–31] in an effort to stay close to the data collected from participants. Codebook thematic analysis entailed deconstruction of the data into isolated fragments using a codebook that delineated the hierarchical relationship between codes. This was then followed by the reconstruction of the data into overarching categories and themes that described ‘what’s going on’ in the data. Two members of our team researchers (LR, JL) undertook an open-coding process to generate first impressions that informed an exhaustive codebook. The coding process began with each team member developing codes independently and then reaching consensus as a team on the codes to include in the codebook. The codebook was then systematically applied for line-by-line coding of all transcripts after agreement was reached between the two coders. Five additional researchers (MBW, AV, ME, KS, RS) participated in the thematic analysis which entailed comparing and contrasting coded data, categorizing similar ideas, grouping categories, and identifying distinct themes. [29] Quotes that represented the emergent themes were carefully selected as a team, in an attempt to accurately convey participants experience and give voice to the issues and challenges being discussed.

Analytic rigor was enhanced by consulting amongst the researchers throughout the analysis process, exercising reflexivity (discussing and journaling the study team’s own biases) by having regular team meetings to share our understandings of the data, ask questions, and discuss how our own lived experiences may be impacting data interpretation. [27] To support data sharing and enhance the reproducibility of the results our protocol, consent forms, demographic questionnaires, interview guides, and detailed codebooks with code summaries have been deposited into a public data repository [https://doi.org/10.5683/SP3/MBALNF]. Our team also adhered to COREQ reporting guidelines (see Appendix A for COREQ checklist). [32]

## Results

In total, we interviewed 67 participants (n = 52 PWLC and n = 15 caregivers). Of note, two of the PWLC also reported being a caregiver to someone else that had Long COVID. On average, PWLC were 50.4 years old and 71.2% self-identified their gender as female and white (86.6%). Furthermore, most participants were educated and married/common-law (65.4%). Caregivers were on average 50.0 years old and 46.7% self-identified as female and white (80.0%). Across participants, we had representation from all provinces and territories except for Prince Edward Island, Nunavut, and Northwest Territories. (See Table 2 for a comprehensive overview of each stakeholder group’s characteristics).

**Table 2:** Overview of demographic information for people with long COVID.

Characteristic	People with Long COVID (n = 52)	Caregiver (n = 15)
Age in years (mean, SD)	50.4 (10.5)	50.0 (14.4)
Sex assigned at birth		
Female (n, %)	39 (75)	7 (46.7)
Male (n, %)	13 (25)	7 (46.7)
Unknown	0 (0.0)	1 (6.7)
Gender Identity		
Female (n, %)	37 (71.2)	7 (46.7)
Male (n, %)	13 (25)	7 (46.7)
Gender queer/nonconforming (n, %)	2 (3.9)	1 (6.7)
Province/Territory		
Yukon (n, %)	1 (1.9)	0 (0.0)
British Columbia (n, %)	10 (19.2)	4 (26.7)
Alberta (n, %)	8 (15.4)	3 (20.0)
Saskatchewan (n, %)	3 (5.8)	0 (0.0)
Manitoba (n, %)	3 (5.8)	1 (6.7)
Ontario (n, %)	15 (28.9)	6 (40.0)
Québec (n, %)	6 (11.5)	0 (0.0)
New Brunswick (n, %)	1 (1.92)	1 (6.7)
Nova Scotia (n, %)	4 (7.7)	0 (0.0)
Newfoundland & Labrador (n, %)	1 (1.9)	0 (0.0)
Ethnicity		
White (n, %)	45 (86.5)	12 (80.00)
Asian – South (n, %)	1 (1.9)	1 (6.67)
Asian – East (n, %)	1 (1.92)	0 (0.0)
Chinese (n, %)	0 (0.0)	1 (6.67)
Latin American (n, %)	2 (3.9)	0 (0.0)
Black – Caribbean (n, %)	0 (0)	1 (6.67)
Mixed (n, %)	3 (5.8)	0 (0.0)
Marital status		
Married/common law (n, %)	34 (65.4)	13 (86.7)
Single (n, %)	8 (15.4)	1 (6.7)
Divorced/separated (n, %)	8 (15.4)	1 (6.7)
Widowed (n, %)	2 (3.9)	0 (0.0)
Highest level of education		
Some high school (n, %)	1 (1.92)	2 (13.3)
High school diploma (n, %)	1 (1.92)	0 (0.0)
Some university (n, %)	4 (7.7)	0 (0.0)
University (n, %)	23 (44.2)	4 (26.7)
Some college (n, %)	2 (3.9)	0 (0.0)
College (n, %)	12 (23.1)	4 (26.7)
Graduate program (n, %)	9 (17.3)	5 (33.3)
Employment status		
Part time/casual (n, %)	3 (5.77)	1 (6.7)
Full time (n, %)	11 (21.15)	6 (40.0)
Disability (n, %)	11 (21.15)	0 (0.0)
Medical leave (n, %)	6 (11.54)	0 (0.0)
Student (n, %)	1 (1.92)	1 (6.7)
Self-employed (n, %)	7 (13.46)	2 (13.3)
Retired (n, %)	6 (11.54)	4 (26.7)
Not employed (n, %)	5 (9.62)	1 (6.7)
Disability/part-time (n, %)	1 (1.92)	0 (0.0)
Care leave (n, %)	1 (1.92)	0 (0.0)
Family income below taxes last year		
$0–19,999 (n, %)	3 (5.77)	1 (6.7)
$20,000–59,999 (n, %)	12 (23.1)	5 (33.3)
$60,000–99,999 (n, %)	12 (23.1)	2 (13.3)
$100,000+ (n, %)	16 (30.8)	6 (40.0)
Prefer not to say (n, %)	6 (11.5)	1 (6.7)
Unknown (n, %)	3 (5.8)	0 (0.0)

PWLC and caregivers shared their experiences of navigating day-to-day life while managing persistent Long COVID symptoms. During data collection and analysis, it became evident that PWLC and caregivers experienced disruptions to their pre-established roles, identities, and envisioned futures, which caused them a great deal of stress. Their experiences highlighted a number of disruptions resulting from the episodic nature of Long COVID and the lasting impacts it had on both their physical and psychosocial well-being. Below we describe three key themes that capture both PWLC and caregiver’s conceptualizations of the disruptions endured, the impact on their daily lives, and how they have adapted and/or managed such disruptions and associated stresses.

### Theme 1: Deviation from perceived ‘normalcy’ and accepting life changes

PWLC and caregivers explained that the experience of Long COVID had changed their sense of normalcy. Many participants felt they were no longer able to perform or participate in previous familial, social, and workforce roles or accomplish associated responsibilities. Participants expressed a deep love and longing for their ‘prior lives’ and this underpinned sentiments that they were no longer “*living my normal everyday life like I was before*” (PWLC01 [female, age 51]). In turn, nearly all participants talked about wanting “*to get back to normal life*” (PWLC03 [female, age 56]), which for them entailed returning to their jobs, participating in meaningful activities, and doing more than just “*surviving*” (PWLC01 [female, age 51], PWLC05 [female, age 50], PWLC11 [male, age 35]). However, participants recognized that they could not necessarily know *“how I am going to be able to do the things that I used to do*” (PWLC20 [female, age 56]). Although biographical disruptions were discussed most prominently by PWLC, caregivers alluded to a ripple effect and commented that “*it’s not just [PWLC] that had her life destroyed, but it’s me as well*” (CG03 [male, age 53]) as they had to quickly adapt to a new caretaking role.

In light of this realization, participants talked at length about life needing to take on a new course and accepting that “*anything that we had planned to do in the future is no longer possible*” (CG03 [female, age 53]). Some of these things included spending time with their family, continuing to progress in their careers, and engaging in other meaningful activities (volunteering in the community, travelling abroad, training for triathlons etc.). Some participants came to this realization quickly while many others talked about wondering “*how long is it going to take me to get back to normal?*” (CG14 [female, age 64]) and “*holding on and hoping that we would ride it out*” only to eventually realize “*it’s not really going anywhere*” (CG06 [female, age 33]). While some participants held onto the belief that “*this is not a permanent thing*” (CG06 [female, age 33]) and refused to believe “*that I will stay like that*” forever (PWLC15 [female, age 49]), other participants had come to “*understand my limitations”* (PWLC03 [female, age 56]) and that “*this is my new life”* (PWLC18 [male, age 45]). For those participants, this meant focusing on the progress they were making by “*looking at the small wins*” (CG05 [female, age 46]) and taking “*life day-by-day*” (PWLC05 [female, age 50]).

### Theme 2: Loss of identity, independence, and meaning

Many people with Long COVID described personal experiences with losing their former identities as they were not able to participate in previous familial, occupational, or social roles that often help people solidify their individuality. Many participants shared the sentiment that they were not who they used to be and that the “*hardest thing is trying to come to terms with [the fact that] I may never be that person again*” (PWLC06 [female, age 58]). This affected their sense of self and changed how they felt about and understood their own personhood. People shared that in losing aspects of their vocational identity, they felt as if they had to *grieve* that part of themselves as well as navigate financial challenges. Caregivers expressed similar feelings, saying “[it’s] *a huge emotional toll itself, because all of a sudden. It’s that real concrete realization that my livelihood has gone, that’s a very difficult emotional concept to accept…I can’t do the normal things that I’m used to doing, that’s a huge piece*” (CG010 [male, age 48]). Caregivers also felt as if they had “*been erased from the world*” (CG03 [male, age 53]) and that “*both of us* [PWLC and caregiver] *have a longing for the person that they used to be, and the person I used to be*.” (CG12 [non-binary, age 43])

A substantial part of participants’ identity was also tied to their occupation as employment can often give people meaning and purpose in their lives. People frequently mentioned that they “*want to work. I loved my work*” (PWLC03 [female, age 56]) because “*that is who I am… I just want normalcy. I want to get back my life*” (PWLC01 [female, age 51]). Some PWLC shared feeling like they “*had this career which I love, and all of a sudden, overnight, everything’s just snatched away”* (PWLC40 [male, age 57]) as they felt they were “*going to lose everything I’ve worked my whole life for*” (PWLC03 [female, age 56]). PWLC felt motivated to return to work because they “*want to be able to be productive*” (PWLC13 [male, age 64]) or they felt like it “*gives me my independence. It gives me security”* (PWLC03 [female, age 56]) so that they can “*live more purposeful*” (PWLC05 [female, age 50]). However, PWLC described the impacts on their physical health and cognition as severely impacting their ability to work, saying “*if my brain’s not working properly, how can I work?*” (PWLC10 [female, age 53]). Many PWLC also expressed financial concerns of not being able to work, saying “*it was costing me, at least, two to three thousand dollars a month while I was out of work to deal with this and to get healthy so that I could go back to work.*” (PWLC42 [female, age 53]). Not being able to contribute financially, often made PWLC feel that they had become a financial burden on their family and loved ones. Similarly, caregivers also felt their employment afforded them a sense of ‘normalcy’ that balanced their new caregiving roles. For example, caregivers talked about work as giving “*a bit of a reprieve*” (CG06 [female, age 33]) away from their added responsibilities. Furthermore, caregivers commonly discussed needing to work to compensate for the loss of income by their family member with Long COVID, “*otherwise we’re not going to make bills*” (CG06 [female, age 33]).

The toll that Long COVID symptoms took on participants’ physical, mental, and cognitive health often rendered them unable to accomplish their previous responsibilities (e.g., activities of daily living such as personal care). PWLC commonly reported feeling the loss of independence as they needed to rely on others (e.g., caregivers) to help them complete instrumental daily tasks such as household chores and errands. This sense of loss was amplified amongst PWLC who felt they had always “*been independent*” (PWLC03 [female, age 56], PWLC43 [female, age 47], CG02 [female, age 52], CG03 [female, age 53]) throughout their lives, as well as those who were considered themselves to be younger in age (those of working or child-rearing age) and/or had childcare responsibilities. The inability to be as independent as they once had been evoked feelings of inadequacy (e.g., lack of confidence when completing ADLS, not being able to uphold their previous roles and responsibilities).

Dependency appeared to be felt most intensely in the context of the relationships of PWLC with their family members (most commonly, spouses). As partners took on caregiving roles, they not only became responsible for previously shared tasks, they also had to manage their new caregiving duties (e.g., administering medications, scheduling appointments, etc.). In some cases, this put a strain on their intimacy, as it was not the dynamic they had envisioned prior to Long COVID: “*we entered this marriage really intending to be very independent people. And so we didn’t want to enter this where [PWLC] was going to become my dependent. But [PWLC] is not going to be able to work with what she has right now*” (CG03 [male, age 53]). As a result of the shift in relational dynamics between the PWLC and caregivers, both groups experienced a range of impacts such as a lack of intimacy, feelings of frustration, and managing difficult emotions (e.g., unwanted guilt and resentment). Parent-to-child dynamics also changed as children of PWLC who were still living at home had to also step up as a caregivers for their parents by taking on more of the household duties. Especially, for people who were either divorced or single, they described having to locate alternative support options (e.g., having an their adult children help with household duties, or move back home and provide household income, or paid in-house support, etc.).

Participants also described feeling helpless in their situations, bringing up existential issues and feelings of a loss of meaning and purpose with their lives. Some participants used metaphors to indicate and emphasize that they felt as though they were disappearing when describing what they felt had happened to them as a result of experiencing, or caring for someone with, Long COVID. For example, one caregiver expressed feeling like Long COVID “*erases people from the world*” (CG03 [male, age 53]) as PWLC and caregivers are confined to the safety of their homes, becoming “*kind of invisible*” (PWLC47 [female, age 49]).

Many people with Long COVID also described not knowing or understanding what was happening to them, besides feeling unsupported in health care settings. Such experiences led PWLC to feel helpless, not believing that they could or would get better. This sense of helplessness often manifested as fears about the future. Some PWLC and caregivers expressed that they were afraid they were (or their loved ones were) “*dying*” or “*going to die*” (PWLC17 [female, age 51], PWLC18 [male, age 47], PWLC38 [female, age 51], CG14 [female, age 64]). In some extreme cases, PWLC worried about death but felt such an intense sense of hopelessness that they thought “*if I were to die right now, I really wouldn’t mind. I think [death] has got to be better than this*” (PWLC18 [male, age 45]). However, the majority of future-facing fears revolved around the possibility of always having a debilitating condition that they could do nothing about for the rest of their lives. The fact that participants “*didn’t know whether* [Long COVID] *is a permanent condition now*” (CG07 [male, age 81]) created intolerable uncertainty for people who “*don’t want to live like this*” (PWLC11 [male, age 35]) and caregivers who find it “*hard to just watch someone suffer and not be able to do anything to help*” (CG03 [male, age 53]). PWLC also shared sentiments of “*I don’t think my life is going to be as long as I thought*” (PWLC41 [female, age 59]). The persistence of disabling symptoms made it difficult for participants to envision a return to well-being, leaving many to feel like there was not much to look forward to and not much left they could do with their lives. They expressed a deep sense of emotional pain about “*not doing things that I love to do…it hurts me inside, it digs at me inside*” (PWLC48 [male, age 67]). Loss of meaning in life made it harder to cope and move forward, and not having hope made it especially difficult to “*get up in the morning and…have a purpose*” (PWLC49 [female, age 62]). Several participants elaborated on this sense of futility by saying they felt like they are “*stuck*” (PWLC18 [male, age 45]), “*suffering with something that holds you back from being at your full potential*” (PWLC20 [female, age 66]), and ultimately feeling like they “*can function and be sort of okay if I just do basic things. But I don’t feel like that’s a meaningful existence…it’s not satisfying*” (PWLC47 [female, age 49]).

### Theme 3: Coping by adapting roles and daily activities

People described having to adapt and/or adjust their activities of daily living such that they could manage and navigate COVID’s impact on their general debility (i.e., chronic fatigue, lack of strength, post-exertional malaise, shortness of breath, exceedingly high heart rate from simple tasks, emotional well-being (depression, anxiety), and cognition (brain fog). For most, this meant “*basically adjust[ing] my life according to what I can do and what I can’t do*” (PWLC01 [female, age 51]). This means, activities that people previously would not have given a second thought to (e.g., cooking, cleaning, showering, taking out the trash, getting groceries, or doing the laundry) required time, effort, and planning. This entailed things like “*having to lie down for almost an hour to recover* [after a shower]” (PWLC09 [female, age 53]), “*laying down with a computer to do work*” (PWLC11 [male, age35]), and “*trying to cook….but having to have someone with me*” (PWLC01 [female, age 51]). Governed heavily by energy levels, people noticed that they would have to plan which days they would perform certain tasks in advance to ensure they had adequate time to recover from the imminent ‘crash’ that the exertion would cause. Some participants explained that they “*started looking at it almost as an* [energy] *budget and that’s how I started to structure my day*” (PWLC05 [female, age 50]). In a similar vein, some participants referred to using a ‘spoon theory’ approach whereby a PWLC has a finite number of spoons that symbolize the amount of energy they can expend when engaging in potentially draining activities. For example, “*you’ve got* [for example] *10 spoons for the day*” (CG10 [male, age 48]) and each task takes up a certain number of spoons (e.g., “*so even showering takes up a couple of spoons*” (CG10 [male, age 8]) and you ultimately only have “*so many spoons in a day for tasks you can do*” (PWLC37 [female, age 32]) in order to not over extend themselves and conserve physical and mental energy.

For caregivers, a great deal of biographical disruption was tied to their experiences of taking on a new caregiver identity, while simultaneously losing other previous roles/identities. Caregivers talked about being pulled in many directions at once and how “*my time used to be my own, but it isn’t so much these days*” (CG04 [male, age 69]). Caregivers also expressed that their new roles/responsibilities were taking over every aspect of their life (e.g., managing medical appointments, taking care of family, keeping up with housework) and their “*life was basically consumed by taking care of* [PWLC] *and his needs*” (CG11 [female, age 62]). Like the PWLC, caregivers felt their role “*consumes a lot of the energy that I have in my daily life…it’s hard to feel that I’ve got energy left over to do the things that are healthy for me*” (CG03 [male, age 53]). Although caregivers recognized that they needed to find “balance” with caregiving and other roles, they often ended up de-prioritizing themselves and “*putting a lot of* [personal interests] *aside*” (CG10 [female, age 48]).

## Discussion

This qualitative study was one of the first to explore the stressful disruptions experienced by both PWLC and caregivers and how such disruptions have impacted their health, well-being, and navigation of daily life. Our findings elucidate that Long COVID fractures the sense of normalcy and envisioned futures of both the person with Long COVID and their caregivers. In turn, feelings of grief ensue due to the loss of personhood, agency, meaning, and purpose in life. Although finding their own new normal would seemingly be beneficial, participants had not yet reached this stage of acceptance, instead focusing on discussions surrounding the adaptations and self-management techniques they utilized to cope with Long COVID.

### The experience of long COVID-related disruptions

The disruptions experienced by PWLC and caregivers forced both groups to re-examine their understandings of their futures. Our participants’ narratives highlight that similar to disruptions due to other chronic illnesses, Long COVID-related disruptions were stressful in as far as they fractured many “taken-for-granted” assumptions and behaviors that shape their daily lives. [33] Participants in our study described how Long COVID rendered them unable to carry out activities of daily living (ADLs), fulfill their responsibilities, and work as they used to. This disruption extended to caregivers whose new responsibilities changed their ability to maintain their daily routines. Due to these disruptions and the associated stress, grief ensued over their lost identities, personhood, independence, and agency as they face.

Some disruptions were more visible or specific to either PWLC or caregivers (e.g., PWLC often experienced more disruption in their employment and career identities, while caregivers experienced more disruption in the role-transition aspect). Yet, many disruptions experienced by PWLC and caregivers overlapped. For example, both PWLC and caregivers felt that the ‘shared future’ they had envisioned was disrupted. This was especially true for individuals in intimate relationships with each other. Thus, dyadic support interventions (i.e., those that target PWLC and caregivers as a pair) may hold unique value in the context of Long COVID as they can help PWLC and caregivers *co-navigate* unexpected disruptive changes together, allow them to better-integrate disruptions into their *collective* biographies, and re-examine what their future *together* may look like. [16,17,34] Future studies that interview PWLC and their caregivers as a dyad would meaningfully contribute to both the creation and evaluation of dyadic interventions for this population.

### Coping with and adapting to stressful disruptions

The prevalent sentiment from participants in our study was that Long COVID (and associated disruptions) left them feeling ‘stuck’, helpless, and hopeless. This hopelessness was exacerbated by the lack of a definitive prognosis for Long COVID. In turn, PWLC and caregivers experienced substantial declines in their mental and emotional health and well-being. This was characterized by stress, uncertainty, grief, and disengagement from relationships and meaningful activities due to the unpredictable symptoms of Long COVID (e.g., leisure, spending time with family/friends, self-care activities, etc.). Caregivers experienced added burdens due to the emotional distress of helplessly witnessing their loved ones struggle. Collectively, these findings emphasize that a bio-psychosocial approach is pressingly needed to mitigate the stressful and deleterious impact Long COVID-related disruptions have on emotional health and well-being for both PWLC and caregivers.

This includes providing more psychological support that helps PWLC and caregivers cope with the emotional challenges and grief associated with the disruptions they have experienced. [35] For example, interpersonal therapy would be helpful to look at the domains of loss experienced by PWLC and caregivers as they navigate a major role transition and broaden their social supportive networks. [36] Family counselling can also help to address changing relational dynamics, loss of intimacy, and threats to relationship breakdowns resulting from stressful disruptions. [37] Family/marital counselling can help PWLC and caregivers maintain a semblance of ‘connection and mutuality’ with each other, enable them to symbolically reconstruct memories to help cope with the emergent disruptions, and help them establish and maintain boundaries with each other and others in their social circle. [16,38,39] Additionally, vocational rehabilitation (e.g., workplace accommodations, job training) can aid PWLC in exploring return to work options. [40] This type of holistic care and Long COVID management is best-supported by a multi-disciplinary, collaborative team approach that is emblematic of bio-psychosocially informed rehabilitation that address the physical, mental, cognitive, and social aspects of wellbeing (e.g., speech therapy, occupational therapy, physiotherapy, psychology or psychotherapist, etc.). [41]

### Moving towards a “New Envisioned Future”

While many participants shared similar sentiments of wanting to ‘get back to normal’ and needing to adapt and/or adjust their activities of daily living to lessen the burdens and impacts Long COVID has on their lives (e.g., spoon theory), participants did widely discuss how they were re-configuring their identities alongside their experience of Long COVID/disability. This finding points to the likelihood that most participants had not yet reached a point where they could take active steps towards constructing a ‘new envisioned future’ reflective of the realities of Long COVID. As part of rehabilitation and self-management efforts, PWLC and caregivers can be guided towards healthy behaviours that help them not only *cope* with their current circumstances, but importantly, also *hope* for their futures. Compassionate and supportive approaches that address the physical, emotional, and social wounds left behind by Long COVID can aid PWLC and caregivers in identifying positive coping strategies and ways of progressing to a ‘new envisioned future’. This could well include: professional grief counseling or therapy that provides a safe space for individuals to express their feelings of loss, sadness, and grief related to the disruptions; narrative therapy practices that explore one’s life and re-frames their experiences by identifying potential room for growth and adaption; as well as expressive therapies such as art therapy, music therapy, and narrative journaling to provide a creative outlet for expressing emotions and processing grief. [42–47] Collectively, these approaches can foster a sense of purpose and direction amidst the disruptions that PWLC and caregivers experience. [48] Future studies could explore the extent to which these interventions are effective by exploring their ability to foster hope and mitigate feelings of hopelessness and helplessness amongst PWLC.

### Nobody left behind–Ensuring caregiver support

While Long COVID most directly impacts PWLC as they navigate the episodic nature of this condition, caregivers also experienced notable disruptions in their own lives. Our study aligns with research on caregiver-specific disruptions in other chronic illnesses (e.g., stroke, cancer, Parkinson’s) that demonstrates caregivers also experience discontinuity with their life course. [16,34,38,39] Caregivers in our study commonly talked about having to ‘pick up the slack’ with household and child care duties while also managing their loved ones’ health concerns—leaving no time for self-care. In this way, the Long COVID caregiving role is ‘all encompassing,’ with caregivers having ‘nothing left in the tank’ after completing caretaking responsibilities. This emphasizes that caregiver-specific supports cannot be overlooked in order to help them navigate their new role while also balancing other responsibilities and aspects of their identity. [49,50] Future research should endeavor to understand what needs may be unique to caregivers and what avenues would be best-suited for ensuring they are supported in their role. For example, providing caregivers with access to respite support can allow them to have much-needed breaks, thereby mitigating burnout and compassion fatigue. [51] Advocating for more funding and resource allocation towards home care so that respite support for caregivers is publically-funded and formally provided by trained professionals (e.g., personal support workers) is needed. [49,51] Having adequate at-home support may also help reduce the risk of relational breakdowns by improving both the well-being of the caregiver and patient [52] and the quality of the patient-caregiver relationship. [51]

## Strengths and limitations

In relation to current research on the psychosocial impacts of Long COVID, our inclusion of caregiver perspectives provides important and unique insight into how their lives are impacted by Long COVID and indeed how Long COVID affects relationships. Additional strengths of our study include achieving a large sample size across a range of geographic locales in Canada which allowed us to capture a breadth of experiences that added depth and richness to the key ideas we identified during thematic analysis. Further, our robust and interdisciplinary study team ensured that diverse perspectives informed our analysis, thereby reducing bias and enhancing the analytic rigor of our work.

While we aimed to recruit participants from varying intersectional identities (e.g., age, sex, race, ethnicity, gender, sexuality, SES, ability or disability status), the majority of our participants self-identified as female and white. This limits our understanding of Long COVID-related disruptions across differing racial and ethnic minorities and members of the 2SLGBTQ+ community. Furthermore, the majority of the participants were well-educated with many holding either a college or a university degree and many identified as being married and/or having a common-law partner. This means that our findings may not be transferable to people who have lower socioeconomic status and less support living in the community. Finally, our study focused on adults’ experiences with Long COVID-related disruptions and thus our findings are likely not transferable to children and youth with Long COVID who may experience unique disruptions relative to the stage of their life course.

## Conclusion

Although research is beginning to identify the types of disruptions commonly experienced by people with Long COVID, the present study advances our understanding by elucidating how Long COVID-related disruptions are co-experienced by the PWLC and caregivers in addition to the disruptions unique to each group. Overall, there was a great deal of alignment in the types of disruptions experience by PWLC and caregivers, with both experiencing impaired health and well-being due to the grief of losing meaningful roles, identities, and envisioned futures. Neither PWLC nor caregivers had reached a point of conceiving of a ‘new normal’, highlighting a potential opportunity for hope- and goal-oriented interventions. Our study also points to the need for bio-psychosocial care for PWLC and caregivers in order to address Long COVID’s impact on the various domains of health and well-being. With appropriate resources and supports put in place, health and social care providers are particularly well-positioned to advance this mission by engaging in and advocating for person- and family-centered approaches to care that address key psychosocial needs. There is a need for more research exploring the psychosocial impacts Long COVID has on the lives of PWLC and caregivers, particularly, longitudinal research that better explores Long COVID experiences over a wider span of time.
