# Correction: Coping with stressful life disruptions due to long COVID: A qualitative study

**DOI:** 10.1371/journal.pone.0336986

**Published:** 2025-11-14

**Authors:** The PLOS One Staff

There are errors in the author affiliations. The correct affiliations are as follows:

Marina B. Wasilewski^1^, Jaylyn Leighton^1^, Logan Reis^1^, Abirami Vijayakumar^1^, Mahala English^1^, Kris Sanchez^1^, Sander L. Hitzig^1^, Michelle L.A. Nelson^2^, Christine Sheppard^3^, Lawrence Robinson^1,4^, Rosalie Steinberg^1,5^, Melody Nguyen^5^, Mark Bayley^4,6^, Nick Daneman^7^, Charissa Levy^8^, Susie Goulding^1^, Chester Ho^9,10^, Robert Simpson^7,9^

**1** St. John’s Rehab Research Program, Sunnybrook Research Institute, Sunnybrook Health Sciences Centre, Toronto, Ontario, Canada, **2** Sinai Health Science of Care Institute; Institute of Health Policy, Management and Evaluation, Dalla Lana School of Public Health, Toronto, Ontario, Canada, **3** FactorInwentash Faculty of Social Work, University of Toronto, Toronto, Ontario, Canada, **4** Sunnybrook Health Sciences Centre, Toronto, Ontario, Canada, **5** UHN Toronto Rehabilitation Institute, Toronto, Ontario, Canada, **6** Sunnybrook Research Institute, Toronto, Ontario, Canada, **7** University Health Network, Toronto, Ontario, Canada, **8** Division of Physical Medicine and Rehabilitation, Department of Medicine, University of Toronto, Toronto, Ontario, Canada, **9** Division of Physical Medicine & Rehabilitation, Department of Medicine, University of Toronto Toronto, Ontario, Canada, **10** Faculty of Medicine & Dentistry, University of Alberta, Edmonton, Alberta, Canada.

The publisher apologizes for the error.
